# Lipschütz ulcer: A case series of non‐sexually acquired genital ulceration in young women

**DOI:** 10.1111/ddg.15815

**Published:** 2025-07-02

**Authors:** Julian Steininger, Michael Eckert, Sophia Lehr, Susanne Abraham, Stefan Beissert, Claudia Günther

**Affiliations:** ^1^ Department of Dermatology Medical Faculty Carl Gustav Carus Technical University Dresden Dresden Germany; ^2^ Outpatient Clinic for Gynecology and Obstetrics Vilsbiburg Germany; ^3^ Department of Dermatology Eberhard Karls University Tübingen Germany

Dear Editors,

Non‐sexually acquired genital ulceration, also referred to as Lipschütz ulcer or ulcus vulvae acutum, is a rare condition characterized by acute, painful ulcers of vulva and vagina.^1^, ^2^, ^3^ This condition represents an important differential diagnosis in distinguishing it from sexually and non‐sexually transmitted infections, autoimmune diseases such as Behçet's disease or Crohn's disease, drug‐induced reactions, and localized manifestations of other systemic conditions.^1^ It has a benign etiology and is primarily managed with analgesics.

This report details two cases of young women who presented with extremely painful vulvar ulcers.

Patient 1 was a 16‐year‐old female who presented with a 2‐day history of vulvar pain. This was preceded by a 4‐day episode of fever (up to 40 °C), sore throat, cough, and submandibular swelling. Upon examination, erythema, swelling, and a 2 × 1 cm hemorrhagic ulcer with fibrin‐coated margins were observed on the right labium majus, along with a 1 cm fibrin‐coated ulcer on the anterior vaginal wall (Figure 1a).

**FIGURE 1:**
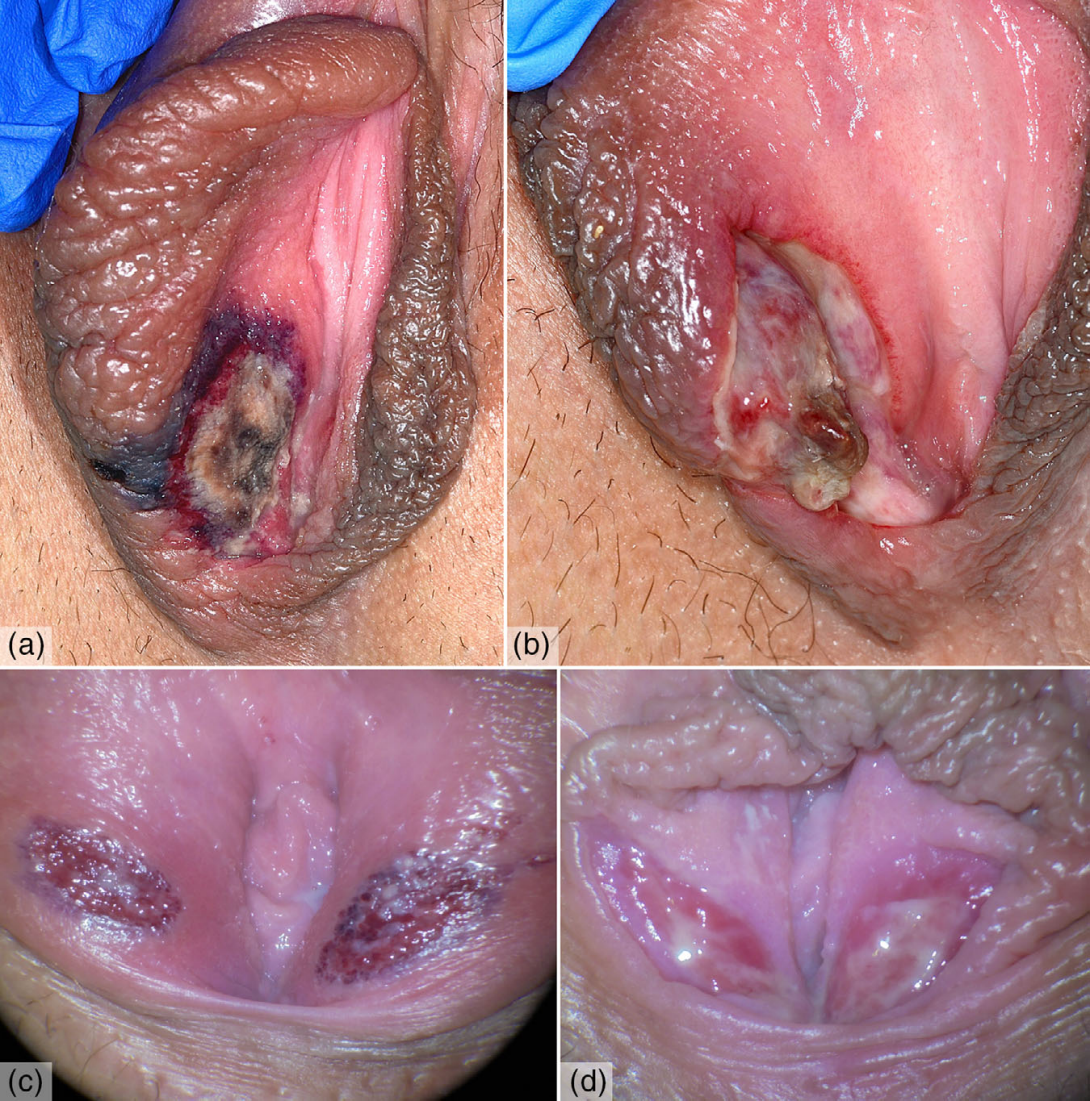
Clinical presentation of two patients with non‐sexually acquired genital ulceration. (a) Painful hemorrhagic and fibrinous ulcer on a swollen right labium majus on admission. (b) Early healing process with fibrinous remodeling 6 days later. (c) Symmetrical erythematous and peripheral bluish‐livid ulcers on both labia minora on admission (magnification x 7.5). (d) Non‐irritated granulation tissue after 4 weeks (magnification x 7.5).

The patient had no prior history of vulvar ulcers. She reported a history of recurrent oral ulcers during childhood and denied any history of sexual activity. Initially suspecting a genital herpes infection, the patient was treated with acyclovir, acetaminophen, metamizole, and polyhexanide gel. Extensive testing was conducted to investigate potential viral, bacterial, and autoimmune etiologies. Serological tests for cytomegalovirus, Epstein‐Barr virus, parvovirus B19, and syphilis were negative, as was PCR testing for mycoplasma. Lesion swabs were negative for herpes simplex virus types 1 and 2. Consequently, antiviral treatment was discontinued, and cefuroxime was started to prevent superinfection. Additionally, testing for relevant autoantibodies, including ANAs, yielded negative results. Histological examination confirmed mixed inflammatory infiltration without vasculitis and the diagnosis of non‐sexually acquired genital ulceration was made. The ulcerations showed fibrinous transformation within a few days (Figure 1b) and healed completely within six weeks without scarring.

Patient 2 was a 21‐year‐old female presenting with symmetrical erythematous and peripheral bluish‐livid ulcers on both labia minora, each with a maximum diameter of 0.8 cm (Figure 1c). There was no evidence of infection or deterioration in her general condition before the onset. The patient's last sexual intercourse was 1 week prior to presentation. Similar to patient 1, viral and bacterial tests were negative, and histology showed non‐specific inflammation. The patient was treated with topical glucocorticosteroids, lidocaine, and analgesics. After 4 weeks, non‐irritated granulation tissue had developed (Figure 1d). At the regular follow‐up 3 months later, complete healing without complications was observed.

Lipschütz ulcers, first described in 1912, typically present as single or multiple vulvar lesions, often accompanied by fever and lymphadenopathy.^2^ The ulcers are usually characterized by a yellow to gray moist base with erythematous margins and are associated with severe pain, which may impair mobility and urination.^3^ Although they can occur at any age, they are most commonly observed in sexually inactive adolescent females.^4^, ^5^, ^6^ Moreover, Lipschütz ulcers can be associated with viral and bacterial infections, particularly acute Epstein‐Barr virus infection.^4^, ^5^, ^6^, ^7^, ^8^, ^9^ Consequently, influenza‐like or pharyngeal symptoms frequently precede the appearance of the ulcers.

The exact pathogenesis remains unclear, and histopathology is generally non‐specific,^6^, ^10^ serving primarily to exclude vasculitis or malignancy. Lipschütz ulcers are self‐limiting and typically resolve within 10 days to 6 weeks without scarring. Standard treatment therefore is supportive, comprising antiseptics, analgesics, and measures designed to facilitate wound healing. In the past, systemic corticosteroids were often administered to accelerate the healing process, but new data suggest that this approach does not provide any significant benefit.^9^

Retrospective data from 110 women with genital ulcers showed that 30% were diagnosed with Lipschütz ulcers. In addition, one‐third of the affected patients reported at least one previous similar episode.^6^ This highlights the importance of considering Lipschütz ulcers in the differential diagnosis of genital ulcers, particularly in sexually inactive young women, to avoid unnecessary treatment and ensure appropriate management.

It is important to note that serological testing may be limited, particularly during the very early stages of infection, due to the window period in which antibodies may not yet be detectable. Therefore, in cases of inconclusive findings, repeat testing or alternative diagnostic approaches should be considered. In our patients, the combination of an extensive diagnostic workup, clinical presentation, and histological findings strongly supported the diagnosis of Lipschütz ulcers.

## CONFLICT OF INTEREST STATEMENT

None.
